# Divergent leukaemia subclones as cellular models for testing vulnerabilities associated with gains in chromosomes 7, 8 or 18

**DOI:** 10.1038/s41598-021-00623-w

**Published:** 2021-10-27

**Authors:** Michael Maher, Jeannine Diesch, Marguerite-Marie Le Pannérer, Marta Cabezón, Mar Mallo, Sara Vergara, Aleix Méndez López, Alba Mesa Tudel, Francesc Solé, Marc Sorigue, Lurdes Zamora, Isabel Granada, Marcus Buschbeck

**Affiliations:** 1 Cancer and Leukaemia Epigenetics and Biology Program, Josep Carreras Leukaemia Research Institute (IJC), 08916 Badalona, Spain; 2 Program for Predictive and Personalized Medicine of Cancer, Germans Trias i Pujol Research Institute (PMPPC-IGTP), Campus Can Ruti, 08916 Badalona, Spain; 3 Department of Hematology Laboratory, ICO-Hospital Germans Trias i Pujol, Josep Carreras Leukaemia Research Institute (IJC), 08916 Badalona, Spain; 4 Microarrays Unit, Josep Carreras Leukaemia Research Institute (IJC), 08916 Badalona, Spain; 5 MDS Group, Josep Carreras Leukaemia Research Institute (IJC), 08916 Badalona, Spain

**Keywords:** Haematological cancer, Acute myeloid leukaemia, Cancer genetics, Biomarkers, Translational research

## Abstract

Haematopoietic malignancies are frequently characterized by karyotypic abnormalities. The development of targeted drugs has been pioneered with compounds against gene products of fusion genes caused by chromosomal translocations. While polysomies are equally frequent as translocations, for many of them we are lacking therapeutic approaches aimed at synthetic lethality. Here, we report two new cell lines, named MBU-7 and MBU-8, that differ in complete trisomy of chromosome18, a partial trisomy of chromosome 7 and a tetrasomy of the p-arm of chromosome 8, but otherwise share the same mutational pattern and complex karyotype. Both cell lines are divergent clones of U-937 cells and have the morphology and immunoprofile of monocytic cells. The distinct karyotypic differences between MBU-7 and MBU-8 are associated with a difference in the specific response to nucleoside analogues. Taken together, we propose the MBU-7 and MBU-8 cell lines described here as suitable in vitro models for screening and testing vulnerabilities that are associated with the disease-relevant polysomies of chromosome 7, 8 and 18.

## Introduction

Haematological malignancies are clonal disorders that originate from somatic mutations in haematopoietic stem and progenitor cells. These mutations often occur in epigenetic regulators and thus affect a variety of downstream processes (reviewed in Maher and Diesch et al.^1^). Cytogenetic alterations are frequent in leukaemia and lymphoma and are detected in 50 to 60 percent of acute myeloid leukaemia (AML) patients^2^. Chromosomal alterations include translocations frequently encoding fusion oncogenes, which can cause functional losses and gains that affect entire chromosomes^3^. Abnormal karyotypes represent powerful prognostic factors^4^. This has led to the inclusion of certain cytogenetic abnormalities in the WHO classification for AML^5^ and their analysis as part of routine diagnostics. Primarily depending on the presence of translocations and gene fusions, AML patients can be stratified into three prognostic classes: favourable, intermediate and unfavourable, according to the aggressiveness of the disease and/or poor response to treatment^6^. For instance, the t(15;17)(q24;q21) translocation is associated with favourable prognosis due to the availability of drugs targeting the resulting PML-RARα fusion gene^7^. On the other hand, other gene fusions are associated with increased leukaemogenic expression. *KMT2A*-*MLLT3,* caused by the t(9;11)(p22;q23) translocation is associated with intermediate prognosis, while the inversion of chromosome 3 leads to *RPN1-MECOM* (inv(3)(q21q26)) and is associated with poor prognosis (reviewed in Lagunas-Rangel et al.^6^). In general, complex karyotypes are usually associated with a poorer outcome. Specific chromosomal losses and gains can also be informative for prognosis; however, they are only in very few cases used to stratify patients for treatment. One example is the loss of the q-arm of chromosome 5 in myelodysplastic syndrome that is a selection criteria for lenalidomide treatment with a high rate of favourable outcomes^8^. Another example is monosomy 7 in AML, which is associated with a poor prognosis and thus requires aggressive chemotherapy and hematopoietic stem cell transplantation^9,10^. A better understanding of how cytogenetic changes relate to drug responses is needed to improve patient stratification and greater therapy success. While clinical trials routinely assess cytogenetic profiles using conventional methods, in vitro models are needed to functionally test associations between cytogenetic alterations and responses to tool compounds and drugs in the pre-clinical phase.

Matched patient-derived cell lines representing different states of aggressiveness of a disease are powerful tools. This includes the B16 melanoma cell line model^11^ and engineered cell lines such as the MBU series for HRAS-driven squamous cell carcinoma^12^. Furthermore, many leukaemia- and lymphoma-derived cell lines have been established harbouring various different cytogenetic abnormalities or particular gene mutations^13^. Genetic drift is often seen in cell lines during long-term cultures. Indeed, karyotypic changes in leukaemia and lymphoma cell lines can be seen as a result of increasing passage numbers^14^. To some extent, these changes reflect the same events of clonal evolution occurring in patients during disease progression^15^. Characterizing the functional effects of these chromosomal changes would help identify vulnerabilities associated with specific acquired genetic abnormalities. This has been exemplified in a recent study performed in 27 strains of the frequently used breast cancer cell line MCF7, which display a multitude of genetic variations and considerably different drug responses^16^. And thus, well-characterized cell lines enable screening for differences in drug sensitivity associated with clinically relevant genetic alterations.

Here, we report the characterization of two divergent leukaemia sub-clones derived from U-937 cells. The two cell lines differed in partial and complete trisomies of chromosomes 7 and 18, respectively, as well as tetrasomy 8p. These cytogenetic differences were associated with a differential drug response suggesting that this pair of cell lines can serve as a model to specifically probe for vulnerabilities associated with the clinically relevant polysomies of chromosomes 7, 8 and 18.

## Results

### Isolation of divergent cell clones that differ in trisomies 7, 8 and 18

**Figure 1:**
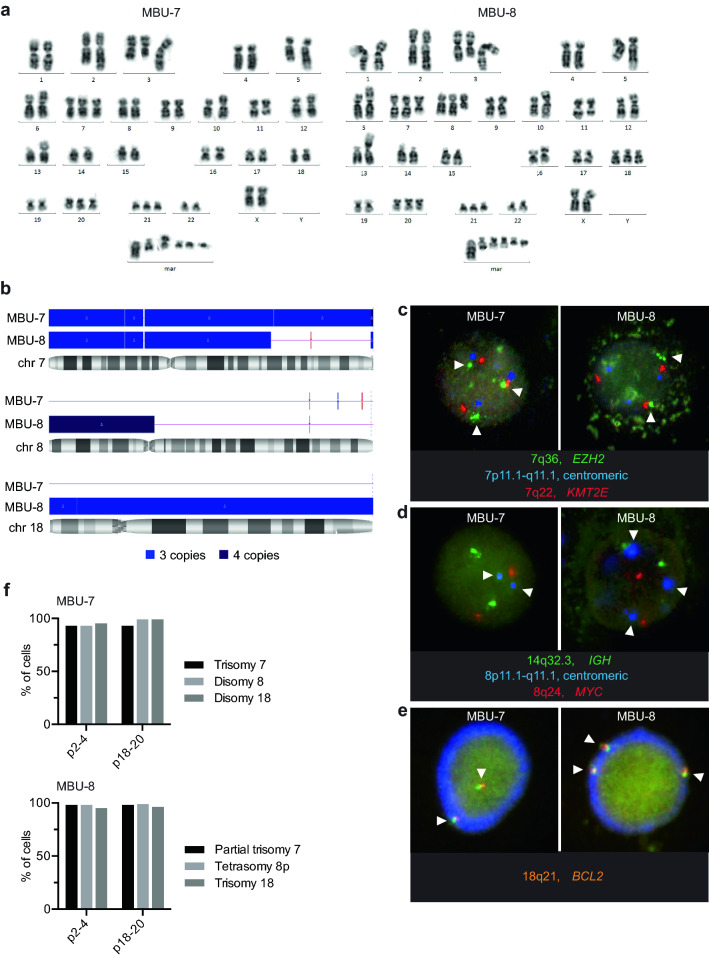
The two cell lines MBU-7 and MBU-8 differ in trisomies 8 and 18 and a partial trisomy of 7. (**a**) Representative karyotype images of MBU-7 and MBU-8 showing cytogenetic differences: trisomy of chromosomes 7 and 18, and tetrasomy 8p. (**b**) Distribution of copy number variations in chromosomes 7, 8 and 18 in MBU-7 and MBU-8 as determined by CytoScan 750 k Array analysis. Blue, gain; red, loss; purple, region of heterozygosity. (**c**) FISH with probes for *EZH2* at 7q36 (green), the centromeric region at 7p11.1-q11.1 (blue) and *KMT2E* at 7q22 (red). (**d**) FISH with probes for *IGH* at 14q32.3 (green), the centromeric region at 8p11.1-q11.1 (blue) or *MYC* at 8q24 (red). (**e**) FISH with probes for *BCL2* at 18q21 (orange). Arrows indicate loci that differ between MBU-7 and MBU-8. (**f**) Quantification of the FISH signal represented in (**c**–**e**) by the Metafer platform in MBU-7 and MBU-8 after 2–4 passages (p2-4) and 18–20 passages (p18-20).

While growing leukaemia cells in suspension over periods of weeks, we noticed a change in the behaviour of the bulk population that was due to the presence and expansion of cells with unexpected characteristics. In order to characterize these cells, we first isolated and expanded single cell clones. We began to characterize two cell lines derived from the expansion of these single cell clones by conventional karyotyping (Fig. 1a). Both cell lines displayed a complex and overall highly similar karyotype. Shared alterations included der(1)t(1;5)(p22;q31.1), der(3)t(1;3)(q21;q26), der(5)t(1;5)(p22;q23.3), add(6)(p25), der(10)t(10;11)(p12;q14)/*PICALM-MLLT10*, del(11)(q22), der(13)t(1;13)(p32;p11.2), del(22)(q13.2q13.3) and unspecific rearrangements involving chromosomes 2q37, 3q12, 6p25, 12p12, and 16p11.1, as well as complete trisomies of chromosomes 20 and 21 (Table 1). Clear differences were the presence of trisomy 18 and a gain in material of chromosome 8 in one of the cell lines, which for this reason we termed MBU-8. We termed the other cell line MBU-7 as it had a complete trisomy 7, while in MBU-8 part of the q-arm of a third chromosome 7 was lacking. These major differences in the amount of genetic material from chromosomes 7, 8 and 18 has further been confirmed using CytoScan 750 K array-based technology (Fig. 1b and Supplementary Table S1). Interestingly, the array-based method indicated that the additional fragment of chromosome 8 contained two fused p-arms making it a partial tetrasomy 8. The array further provided additional resolution and helped to improve the karyotype formula, thus substantiating that these cell lines possess an overall complex karyotype (Supplementary Fig. S1 and Supplementary Data S1).

**Table 1:** Karyotype of MBU-7, MBU-8 and U-937.

Cell line	Karyotype
MBU-7	56<3n>,XX,− Y, − 1,der(1)t(1;5)(p22;q31.1),− 2,add(2)(q37),add(3)(q12), der(3)t(1;3)(q21;q26),− 4,− 5,der(5)t(1;5)(p22;q23.3),− 6,add(6)(p25),− 8,− 9,− 10, der(10)t(10;11)(p12;q14),− 11,del(11)(q22),− 12,add(12)(p12),− 13, der(13)t(1;13)(p32;p11.2),− 14,− 15,− 16,add(16)(p11.1),− 17,− 18,− 19,− 22, del(22)(q13.2q13.3), + 6mar
MBU-8	58<3n>,XX,− Y, − 1,der(1)t(1;5)(p22;q31.1),− 2,add(2)(q37),add(3)(q12), der(3)t(1;3)(q21;q26),− 4,− 5,der(5)t(1;5)(p22;q23.3),− 6,add(6)(p25), del(7)(q31.1q36.3),i(8)(p10),− 9,− 10,der(10)t(10;11)(p12;q14),− 11, del(11)(q22),− 12,add(12)(p12),− 13,der(13)t(1;13)(p22.3;p11.2),− 14,− 15,− 16, add(16)(p11.1),− 17,− 19,− 22,del(22)(q13.2q13.3), + 6mar
U-937 (source: DSMZ)	63(58–69), XXY, t(1;12)(q21;p13),− 2,− 4,der(5)t(1;5)(p22;q35), − 6, + 7, − 9,add(9)(p22),t(10;11)(p14;q23),i(11q),i(12p),add(16)(q22),add(19)(q13), − 20,− 21, + 3mar
U-937 (source: MacKinnon et al.^31^)	62, XX, − Y, del(1)(q12), + der(1)t(1;5)(p22;q31.1),del(2)(p11.2), + der(2)dup(2)(q24.1q33.1)del(2)(q33.1),del(3)(q13.33q24), + psu dic(3;1)(q25.1;p11.1),der(5)t(1;5)(p22;q23.3), + der(5)t(5;13)(q11.2;q14.11)del(5)(q11.2q11.2), + der(6)t(2;6)(p13.2;p22.1, + 7, + dup(7)(p15.3p15.1), + 8,der(10)t(10;11)(p12.31;q14.2)t(10;10)(q23.33q25.2),der(11)t(10;11)(p12.31;q14.2), + der(11)(16pter- > 16p11.2::11p11.12- > 11q12::11q24.12- > 11q24.2::20q11.21- > 20q11.21::20p12.3 > 20pter), + 12, + 15,der(16)t(4;16)(p13;p12.2)del(4)(p14p14)del(4)(p15.1p15.1)del(4)(p15.31p16.1), + 18, + 19,der(20)(20pter- > 20p12.2::15q14- > 15q25.3::20p11.22- > 20q11.21::20p11.21- > 20p11.21:), + 21, + 22 [21]/63,idem, + der(6)del(6)(p21.31)amp(6)(p21.31)dup(6)(p21.31p12.2), del(7)(q22.1q34)[37]/60,idem,der(7)t(6;7)(q27;q21.12), − 12, − 22[13]

Fluorescence in situ hybridization (FISH) confirmed these differences. While both cell lines showed three copies of the centromeric region of chromosome 7 and the locus 7q22, the locus 7q36 containing the *EZH2* gene was only present twice in MBU-8 (Fig. 1c). In line with two additional copies of 8p fused at the peri-centromeric region, we detected a third signal for the centromeric region of chromosome 8 in MBU-8 but only two copies of the 8q24 portion of chromosome 8 containing the *MYC* gene locus (Fig. 1d). Finally, we confirmed the trisomy 18 in MBU-8 using a FISH probe targeting the locus of *BCL2* on 18q21 (Fig. 1e). To examine the cytogenetic stability of MBU-7 and MBU-8 over an extended period of time, we repeated the FISH analysis after 18–20 passages and could not observe any significant differences in the chromosomes assessed (Fig. 1f). This analysis showed that both cell lines are sufficiently stable during a time window that makes them suitable for comparative experimental studies.

In summary, we isolated two cell lines with highly complex karyotypes that, at first glance, did not resemble any other previously described cell line. Importantly, the two cell lines differed in the number of copies of chromosomes 7, 8 and 18.

### MBU-7 and MBU-8 are divergent subclones of U-937

**Figure 2:**
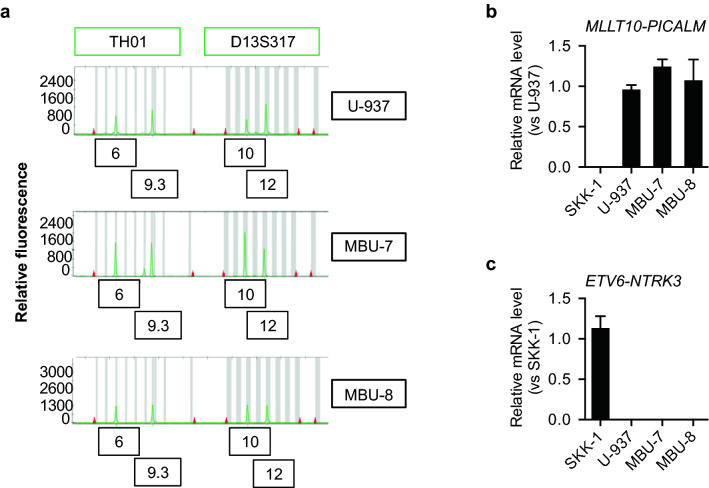
MBU-7 and MBU-8 are derived from U-937 cells. (**a**) Representative electropherograms of the microsatellite loci TH01 and D13S317 measured by STR profiling in U-937, MBU-7 and MBU-8. The numbers in the boxes represent the amount of microsatellites per locus. (**b**) Relative expression of the fusion gene *PICALM-MLLT10* by RT-qPCR in SKK-1, U-937, MBU-7 and MBU-8. Data represent the mean ± SEM of three independent experiments. (**c**) The expression of the SKK-1 specific fusion gene *ETV6-NTRK3* was analysed as in (**b**).

As an alternative approach to identify the origin of these new cell lines, we analysed a panel of short tandem repeats (STR). Profiling of STRs is a widely used forensic technique to identify human individuals by unique signatures based on the allelic number of 16 highly conserved microsatellite sequences^17^. This method has found an important application in the identification of human-derived cell lines whose profiles are collected and stored in the publicly available Cellosaurus database from the Swiss Institute of Bioinformatics^18^. The profiles of MBU-7 and MBU-8 almost perfectly matched the U-937 cell line (Table 2). We confirmed the almost perfect match by repeating the STR analysis with U-937 cells and by visually comparing the profiles. For instance, the short tandem repeat D13S317 was present in 10 and 12 copies in MBU-7, MBU-8 and U-937 (Fig. 2a). Similarly, the repeat TH01 showed 6 and 9.3 copies in all three cell lines (Fig. 2a).

**Table 2:** STR profiles of U-937, MBU-7 and MBU-8.

	U-937	MBU-7	MBU-8
D8S1179	12, 13	12, 13	12, 13
D21S11	27, 29	27, 29	27, 29
D7S820	9, 11	9, 11	9, 11
CSF1PO	12	12	12
D3S1358	16	16	16
TH01	6, 9.3	6, 9.3	6, 9.3
D13S317	10, 12	10, 12	10, 12
D16S539	12	12	12
D2S1338	17, 20	17, 20	17, 20
D19S433	14, 16	14, 16	14, 16
VWA	15	14, 15	14, 15
TPOX	8, 11	8, 11	8, 11
D18S51	13, 14	13, 14	13, 14
AMEL	X	X	X
D5S818	12	12	12
FGA	22,25	22, 25	22, 25

U-937 is a human haematopoietic monoblastoid cell line originally established from a 37-year old male patient with diffuse histiocytic lymphoma^19^, which is a rare, very aggressive type of non-Hodgkin’s lymphoma. Besides their popular use as a differentiation model, U-937 cells are well-studied for the translocation t(10;11)(p12;q14) resulting in a *PICALM-MLLT10* fusion gene^20^. Gene fusions are a common characteristic of both myeloid and lymphoid leukemias and occur due to chromosomal translocations^21^. The resulting fusion genes frequently act as disease-driving oncogenes and have been investigated as part of diagnostic protocols across different leukemias due to their disease subtype specificity^22,23^. As shown in Fig. 2b, we could detect *PICALM-MLLT10*, expression in MBU-7, MBU-8 and U-937, but not in the secondary acute myeloid leukaemia (AML) cell line SKK-1 that we included as a control. Conversely, SKK-1, but not MBU-7, MBU-8 or U-937 cells expressed the *ETV6-NTRK3* gene fusion (Fig. 2c) that is caused by a t(12;15)(p13;q25) translocation^24^.

To define the mutation profile of MBU-7 and MBU-8, we sequenced a previously described panel of 32 genes related to myeloid disorders^25,26^. Three pathogenic variants of *WT1*, *TP53* and *PTPN11* were observed in both subclones with similar variant allele frequency (Table 3). U-937 have been reported to express mutant p53^27^, do not express detectable levels of WT1^28^ and harbour a *PTPN11* mutation (G20R)^29^.

**Table 3:** Variants detected using NGS targeted gene panel^25,26^.

	Gene	Classifi-cation	Type	Chr	Coordinate	Variant	VAF (%)
MBU-7	*WT1*	Class 1	Nonsense	11	32417947	c.1054C > T	49.56
	*TP53*	Class 1	Splice donor + 1	17	7578370	c.559 + 1G > A	99.69
	*PTPN11*	Class 3A	Missense	12	112888162	c.178G > C	51.09
MBU-8	*WT1*	Class 1	Nonsense	11	32417947	c.1054C > T	50.17
	*TP53*	Class 1	Splice donor + 1	17	7578370	c.559 + 1G > A	99.75
	*PTPN11*	Class 3A	Missense	12	112888162	c.178G > C	50.63

Classification^52^: Class 1, relevant in the clinical management of myeloid hemopathies. It has been established as a pathogenic variant in myeloid hemopathies and alters an actionable gene. Class 2, it has been established as a pathogenic variant in solid tumors or non-myeloid hemopathies and alters an actionable gene. Class 3, variant not previously described, affects an actionable gene and in silico predictors or classifies mutations as Class 3A, likely pathogenic; Class 3B, uncertain significance; Class 3C, likely benign.

*VAF* variant allele frequency.

Taken together, these results suggest that MBU-7 and MBU-8 are subclones of U-937 cells. Several groups have reported different karyotypes for U-937, but reports are consistent in that U-937 cells are close to triploid^30,31^. Comparing these karyotypes with the karyotypes of MBU-7 and MBU-8 (Table 1) suggests that the two cell lines have diverged by acquiring additional cytogenetic alterations, such as multiple losses of genetic material. This includes the loss of the Y chromosome, a striking mosaicism given the male origin of the cell line. Overall, the loss of genetic material was more pronounced in MBU-7 cells than in MBU-8.

### Monocytic morphology is accompanied by a shared immunoprofile

**Figure 3:**
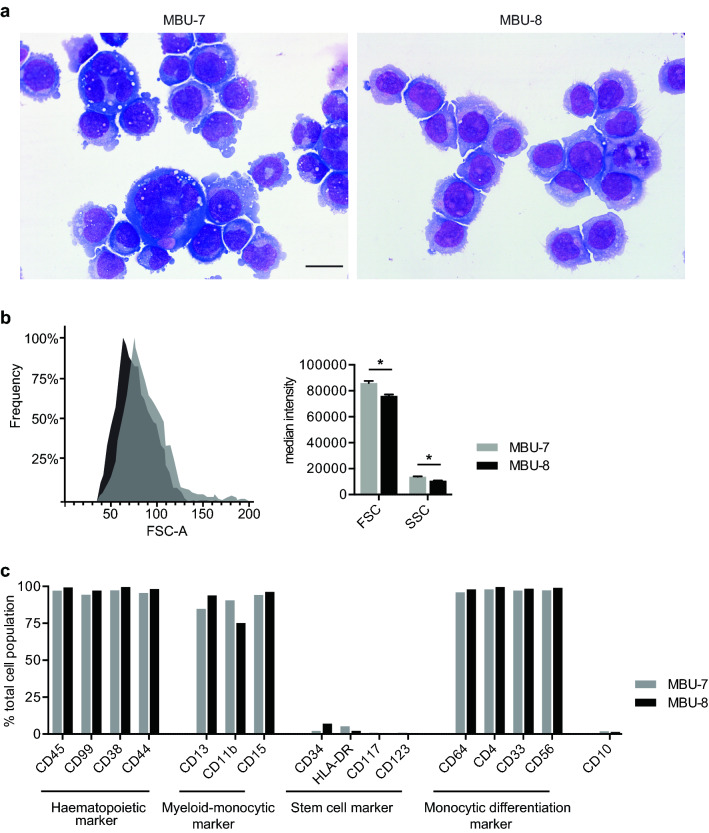
Monocytic morphology of MBU-7 and MBU-8 is accompanied by a shared immunoprofile. (**a**) Microscopy images of MBU-7 and MBU-8 after staining with May–Grünwald Giemsa solution. Scale bar, 10 µM. (**b**) Representative histogram (left) and quantification (right) of cell size of MBU-7 and MBU-8 assessed by flow cytometry (FSC, forward scatter; SSC, side scatter). Data represent the mean ± SEM of three independent experiments. Statistical analysis was performed by Student’s T-test, *p-value < 0.05. (**c**) Immunophenotype as determined by assessing the surface marker expression by flow cytometry in MBU-7 and MBU-8 subclones.

Phenotypically, U-937 cells resemble blast cells of the monocytic lineage^19^. In both MBU-7 and MBU-8, we observed a similar cell morphology resembling monocytic cells (Fig. 3a). Within both populations, cells varied in size and maintained the relationship in size between nucleus and cytoplasma. The nuclei had an irregular contour and lax chromatin with the presence of one or more evident nucleoli. Some cells displayed nuclear herniations, which are abnormal protrusions of the nucleus. The extended cytoplasm was basophilic and, in a subset of cells, presented granulation and vacuoles. Interestingly, in addition a majority of cells with the above described characteristics, both cell lines contained some particularly large cells that were multinucleated and had hyperbasophilic cytoplasm (Fig. 3a). These cells were more frequently seen in MBU-7 than MBU-8, which could be confirmed by measuring the forward and side scatter (FSC and SSC) in flow cytometry (Fig. 3b). Such large cells are often seen in leukaemia patients with complex or hyperdiploid karyotype and can indicate a more aberrant phenotype. Both cell lines were negative for myeloperoxidase, which is a marker for granulopoietic cells (data not shown). In terms of markers for the monocytic-macrophage lineage, both cell lines were positive for alpha-naphthyl butyrate esterase and negative for alpha-naphthyl acetate esterase (data not shown).

We next analysed the immunophenotypes and found that MBU-7 and MBU-8 have very similar surface marker expression (Fig. 3c). Specifically, they were both positive for the hematopoietic markers CD45, CD99, CD38 and CD44, as well as for the myeloid-monocytic markers CD11b, CD13 and CD15. In terms of stem cell markers, MBU-7 and MBU-8 were largely negative for CD34, HLA-DR, CD117 and CD123. Furthermore, virtually all cells expressed monocytic differentiation markers such as CD64, CD4, CD33 and CD56.

In summary, the immunoprofile and morphology of MBU-7 and MBU-8 cells associates them with the monocytic lineage. This is in line with them being derivatives of U-937 cells that, despite having originated from a histiocytic lymphoma patient, have monocytic characteristics. Indeed, U-937 are commonly used to study monocytic differentiation in vitro^32^.

### MBU-7 and MBU-8 differ in drug response

**Figure 4:**
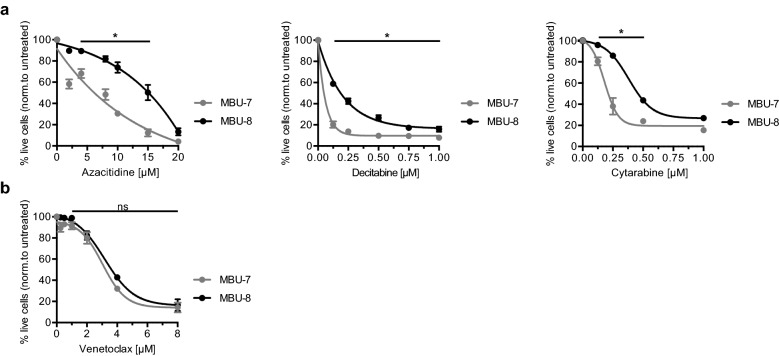
MBU-7 and MBU-8 display different drug sensitivity. (**a**) Percentage of live MBU-7 or MBU-8 cells after 4 days of treatment with indicated concentrations of azacitidine, decitabine or cytarabine. (**b**) Percentage of live MBU-7 or MBU-8 cells after 4 days of treatment with indicated concentrations of venetoclax. (**a**,**b**) Data represent the mean ± SEM of four independent experiments. Statistical analysis was performed using ANOVA. *p-value < 0.01. *ns* non-significant.

Finally, we decided to test the response of MBU-7 and MBU-8 cell lines to a panel of commonly used drugs. Specifically, we have chosen the nucleoside analogues azacitidine, decitabine, and cytarabine. Cytarabine is a DNA toxin and used as a standard chemotherapy drug in AML, acute lymphoid leukaemia (ALL) and non-Hodgkin lymphoma^33^. Azacitidine and decitabine can further act as DNA hypomethylating agents and are the treatment of choice for intermediate- to high-risk MDS patients not eligible for allogenic bone marrow transplantation^34^ and for elderly unfit AML patients^35^. We also included venetoclax, an inhibitor of the apoptosis regulator Bcl2^36^, which is currently being approved for the treatment of an increasing number of haematopoietic malignancies (https://www.fda.gov/drugs/resources-information-approved-drugs/fda-approves-venetoclax-cll-and-sll). Specifically, we have determined the cell viability of MBU-7 and MBU-8 after four days of treatment. MBU-7 cells were significantly more sensitive towards the treatment with all three nucleoside analogues than MBU-8 (Fig. 4a). The biggest difference in response was observed with 0.125 μM decitabine and 0.25 μM cytarabine. Both cell lines were less sensitive to azacitidine but a pronounced difference was observed around 10 μM. In contrast, both MBU-7 and MBU-8 responded in a similar manner to venetoclax suggesting that we are not observing a general propensity of MBU-7 cells to drug-induced apoptosis (Fig. 4b).

Taken together, the cytogenetic differences of MBU-7 and MBU-8 are associated with a differential response to a subset of drugs commonly used for the treatment of haematological diseases.

## Discussion

In our study, we characterized the two cell lines MBU-7 and MBU-8 that we isolated as clones from a suspension culture. The profile of small tandem repeats identified both cell lines as likely derivatives of U-937 cells. This was consistent with a shared mutational profile and a morphology and immunoprofile of monocytic cells. Although the karyotypes of MBU-7 and MBU-8 were different to U-937, they contained some characteristic features shared with U-937 cells such as the translocation t(10;11)(p12;q14) resulting in a *PICALM-MLLT10* fusion gene^20^. Chromosomal instability and loss of heterozygosity has been previously attributed to U-937^37^. Indeed, subclones of U-937 with differences in both small tandem repeats and chromosomal aberrations have been identified following long periods in culture^38,39^. This can explain the divergence in karyotypes of MBU-7 and MBU-8 when compared to U-937. More specifically, U-937 cells have previously been described to have a near triploid karyotype^31^, while MBU-7 and MBU-8 only share trisomies of chromosomes 3, 20 and 21 but differ in complete or partial polysomies of 7, 8 and 18. U-937 cells are derived from a male donor^19^. However, Y chromosome is known to be frequently lost during cell culture^38^. Occasionally this leaves U-937 cells with two X-chromosomes^31^, which we also observed in MBU-7 and MBU-8. Taken together, our results suggest that MBU-7 and MBU-8 are clonal cell lines with divergent karyotypes that spontaneously derived over time from U-937 cells.

MBU-7 and MBU-8 have highly similar characteristics but differ in trisomies 7 and 18 and tetrasomy 8p in the context of a shared complex karyotype. Gains of material of chromosome 8 are frequent in myeloid malignancies. Trisomy 8 is the most frequent cytogenetic alteration in AML occurring in 10 to 15% of patients^40,41^. This includes MDS, where trisomy 8 represents the sole genetic aberration in 11% of cases^42^. Patients with trisomy of chromosome 8 are included in the intermediate-risk group and their prognosis thought to be defined by accompanying aberrations^42^. Trisomy 8 is frequently associated with mutations in driver genes including *RUNX1*, *ASXL1, DNMT3A*^43^, *TET2*^44^, and *IDH1* and *IDH2*^45^. AML with tetrasomy of chromosome 8 has poor prognosis^46^. To our knowledge, tetrasomies of only the p-arm of chromosome 8 have not yet been reported in myeloid diseases. However, it needs to be pointed out that the partial tetrasomy 8 can easily be mistaken to be trisomy 8. Indeed, this would have been our interpretation of conventional karyotyping and FISH data in the absence of quantitative data from the CytoScan array. Importantly, the q-arm carries several cancer-relevant genes such as *FGFR1*, *KAT6A* and *PCM1* (Supplementary Table S1).

In conclusion, MBU-7 and MBU-8 differ in discrete gains of genetic material of chromosomes 7, 8, and 18. The clinical significance of these differences remains to be determined in future studies.

Abnormal and additional chromosomes may have consequences for disease outcome due to changes in gene expression. Compared to MBU-7, MBU-8 had an additional copy of chromosome 18, two additional copies of the p-arm of chromosome 8 but lacked part of a third copy of chromosome 7. We wondered whether these differences in chromosomes would be associated with differences in the response to drugs. Interestingly, our results showed a clear difference between MBU-7 and MBU-8 in the response towards the nucleoside analogues azacitidine, decitabine and cytarabine, with MBU-7 being significantly more sensitive. In this respect, it is interesting to note that 20% of MDS patients that relapsed after decitabine- treatment had acquired additional cytogenetic abnormalities including trisomy 8^47^. Taken together, these observations might indicate a possible link between gains in chromosome 8 and the response of cells to decitabine and related drugs.

Overexpression of the *BCL2* gene on chromosome 18 contributes to MDS by blocking cell death^48^. Even though MBU-7 and MBU-8 differed in the copy number of the *BCL2* gene, they were equally sensitive to the Bcl2-inhibitor venetoclax. From this observation we draw two conclusions: First, MBU-7 cells do not have a general hypersensitivity to treatments that induce cell death. Second, gene copy number of drug targets is not a reliable predictor of drug response. Potential synthetic lethality needs to be assessed on the functional level. The combination of well-characterized cell lines with powerful genetic loss of function screens has the potential to identify novel vulnerabilities associated with distinct genetic and cytogenetic abnormalities in close to unbiased manner^49^.

We propose the here described cell lines MBU-7 and MBU-8 as suitable models for screening for vulnerabilities that are associated with the disease-relevant gains in chromosome 7, 8 and 18.

## Methods

### Cell lines and cell culture

The human cell lines U-937 and SKK-1 were obtained from the Leibniz-Institute DSMZ—German Collection of Microorganisms and Cell Cultures (Braunschweig, Germany). We obtained SKK-1 cells as part of a collaboration with Prof. Hans Drexler that included their detailed cytogenetic and genetic characterization^50^. MBU-7 and MBU-8 have been isolated as single-cell clones and are described here in great detail. Both cell lines will be deposited at DSMZ. All cell lines were cultured in RPMI 1640 (ThermoFisher Scientific, Waltham, MA) supplemented with 10%, 1% Pen/Strep, and 1% l-glutamine at 37 °C in 5% CO_2_. All cell lines were routinely analysed for the presence of mycoplasma.

### RT-qPCR

For real-time quantitative PCR (RT-qPCR), RNA was extracted using the Maxwell RSC simplyRNA Cells Kit (Promega, Madison, Wisconsin) and reverse transcribed with the First strand cDNA synthesis kit (ThermoFisher Scientific). The cDNA was PCR amplified in triplicate using the Fast SYBR green dye on the Applied Biosystems QuantStudio 7 Flex Real-Time PCR System. Relative expression was determined using SKK-1 or U-937 as reference samples, and GAPDH as internal control. The sequences of primers were: GAPDH, forward primer 5′-CGACCACTTTGTCAAGCTCA-3′, reverse primer 5′-TCTTACTCCTTGGAGGCCAT-3′; ETV6-NTRK3, forward primer 5′-CATTCTTCCACCCTGGAAAC-3′, reverse primer 5′-GGCTCCCTCACCCAGTTCTC-3′; PICALM-MLLT10, forward primer 5′-TGAGACCTCCAAACCCCTTT-3′, reverse primer 5′-TCGGCACCATTACCTTCTTC-3′.

### Identifier test

Cells were identified by comparing their short tandem repeat (STR) profiles to their corresponding profiles on the searchable cell line database, Cellosaurus (web.expasy.org/cellosaurus). Genomic DNA was extracted from collected cells using PureLink Genomic DNA Mini Kit (ThermoFisher Scientific) and processed by the Genomics facility (IGTP) using the CLA IdentiFiler Plus PCR Amplification kit (ThermoFisher Scientific) according to the manufacturer’s instructions. Following the PCR, capillary electrophoresis was performed with the amplified microsatellite loci fragments and the allelic ladder, GeneScan 500 LIZ Size Standard, using the Applied Biosystems 3130xl Genetic Analyzer to discriminate alleles that differ by single nucleotides. GeneMapper software (ThermoFisher Scientific) was then used to call alleles according to size and quality based on the selected allelic ladder.

### Cytogenetics

Cytogenetic studies, karyotype and fluorescent in situ hybridization (FISH), were carried out by standard procedures in single cell expanded clones of MBU-7 and MBU-8, at the Cytogenetics Platform, Josep Carreras Leukaemia Research Institute and Institut Català d'Oncologia (ICO).

### Karyotyping

Conventional G-banding metaphases were obtained by standard procedures. In brief, mitotic cells were arrested in metaphase using Colcemid, lysed in hypotonic solution (KCl, 0.075 M), the metaphases fixed with Carnoy’s solution (3 parts of methanol: 1 part of acid acetic glacial) and the G band pattern achieved with Wright dye. Karyotypes for 20 metaphases were analysed for each cell line and described following the International System for Human Cytogenetic Nomenclature (ISCN 2020)^51^.

### Fluorescence in situ hybridization (FISH)

Cells were fixed with Carnoy’s solution and the samples and 5 µL probe simultaneously denatured by heating the slides on a hotplate at 75 °C (2 min). The sides were then incubated in a humidified chamber at 37 °C overnight, washed in 0.4 × SSC (pH 7.0) at 72 °C (2 min), washed in 2 × SSC, 0.05% Tween-20 (pH 7.0) at room temperature (30 s), rinsed in distilled water to avoid crystal formation, air dried. Finally, the cells were counterstained with DAPI and analysed under a fluorescent microscope. Different probes were used to detect several chromosomal abnormalities in MBU-7 and MBU-8. The XL 7q22/7q36 Deletion Probe (MetaSystems, Germany) detects deletions in the long arm of chromosome 7. The IGH/MYC/CEP 8 tri-color dual fusion probe (Abbott, USA) detects the t(8;14)(q24;q32) reciprocal translocation involving the *IGH* and *MYC* gene regions. The *BCL2* break apart probe (Abbott, USA) detects chromosomal rearrangements at the *BCL2* locus on chromosome 18q21. 500 cells per probe have been analysed using the Metafer platform (MetaSystems). The standardised cut-off for probes analyses with this automated system was > 10%.

### Cytoscan array

Genomic DNA was extracted from collected cells using PureLink Genomic DNA Mini Kit (ThermoFisher Scientific) and processed by the Microarray Unit of the IJC. Genomic microarrays were performed with CytoScan 750 K Array from ThermoFisher. Standard protocol and QC guidelines supplied by the manufacturer were followed. The Affymetrix 450 fluidics station and GeneChip Scanner 3000 7G were used to wash, stain and scan the arrays. SNP-array data analysis was performed with Chromosome Analysis Suite Version 4.2 (ThermoFisher Scientific). Data was analyzed using annotations of genome version GRCh37 (hg19). Detailed visual data analysis was performed in all samples, in addition to software-reported alterations. Only copy number (CN) segments with a minimum of 20 SNP/CN-altered markers. Furthermore, germline abnormalities were excluded by comparing findings with control Databases. SNP array data has been deposited in the GEO database under accession number GSE184764.

### Cytology

Cells were immobilized on glass slides at a concentration by cytospin centrifugation (ThermoFisher Scientific) at 300 rpm for 10 min. Once dried, cells were stained with May-Grünwald Giemsa solution (Merck, New Jersey). Cells were further stained with Myeloperoxidase, alpha-naphthyl butyrate esterase and alpha-naphthyl acetate esterase. Cells were mounted with DPX Mountant for histology (Sigma-Aldrich, Misuri) and examined using the microscope Olympus AX70 TRF.

### Immunophenotypic analysis

Immunostaining and flow cytometry analyses were performed at the Clinical Haematology Department at ICO-Hospital Germans Trias i Pujol according to standard procedures with the Navios cytometer (Beckman Coulter, California). The same antibodies are used for diagnostic purposes (see Supplementary Table S2).

### NGS targeted gene panel

Targeted NGS including 32 genes related to myeloid disorders was used. Briefly, DNA was extracted from cell lines and library was prepared using the QIAseq FX DNA library kit (Qiagen, Germany). Target regions were captured and amplified by hybridizing the DNA library with the Myeloid Solution Capture Kit (Sophia Genetics, Switzerland) and, finally, sequenced using MiSeq platform (Illumina, California). The results were analyzed using Sophia DDM platform^25,26^ and interpreted using the classification described in Sukhai et al.^52^.

### Flow cytometric analysis of viability and apoptosis

Following 4 days’ incubation with the indicated inhibitors, cell viability was assessed by flow cytometry of cells stained with 1 μg/mL DAPI (4′,6-Diamidino-2-phenylindole dihydrochloride) (ThermoFisher Scientific), and 100 μM MitoTracker Red CMXRos (ThermoFisher Scientific), using the LSR Fortessa cytometer (BD, New Jersey). Statistical analysis (ANOVA test) was performed using GraphPad Prism software (version 6).

## Supplementary Information

Supplementary Information 1.

Supplementary Information 2.

Supplementary Information 3.

## Data Availability

The CytoScan array data generated in this study have been deposited in the GEO database under accession code GSE184764. Reviewer access: GSE184764: https://www.ncbi.nlm.nih.gov/geo/query/acc.cgi?acc=GSE184764. Password: yrmvqicerjulbel.
