# Supplementary material for: Divergent leukaemia subclones as cellular models for testing vulnerabilities associated with gains in chromosomes 7, 8 or 18
Source: Sci Rep. 2021 Oct 27;11:21145. doi: 10.1038/s41598-021-00623-w (PMC8551338; doi:10.1038/s41598-021-00623-w)
Supplement: Supplementary file 1 — Supplementary Information 1. [file 41598_2021_623_MOESM1_ESM.docx]

**Description of Additional Supplementary Files**

File Name: Supplementary Table S1

Description: Results of the CytoScan 750k array in MBU-7 and MBU-8 cells.
