# Supplementary material for: Divergent leukaemia subclones as cellular models for testing vulnerabilities associated with gains in chromosomes 7, 8 or 18
Source: Sci Rep. 2021 Oct 27;11:21145. doi: 10.1038/s41598-021-00623-w (PMC8551338; doi:10.1038/s41598-021-00623-w)
Supplement: Supplementary file 2 — Supplementary Information 2. [file 41598_2021_623_MOESM2_ESM.pdf]

Supplementary Information

Divergent leukaemia subclones as cellular models for testing vulnerabilities associated with gains in chromosomes 7, 8 or 18

Michael Maher[1,†], Jeannine Diesch[1,2,†], Marguerite-Marie Le Pannérer[1], Marta Cabezón[3], Mar Mallo[4,5], Sara Vergara[3], Aleix Méndez López[3], Alba Mesa Tudel[3], Francesc Solé[4,5], Marc Sorigue[3], Lurdes Zamora[3], Isabel Granada[3], Marcus Buschbeck[1,2,#]

1 Cancer and Leukaemia Epigenetics and Biology Program, Josep Carreras Leukaemia Research Institute (IJC), 08916 Badalona, Spain

2 Program for Predictive and Personalized Medicine of Cancer, Germans Trias i Pujol Research Institute (PMPPC-IGTP), Campus Can Ruti, 08916 Badalona, Spain

3 Department of Hematology Laboratory, ICO-Hospital Germans Trias i Pujol, Josep Carreras Leukaemia Research Institute (IJC), 08916 Badalona, Spain.

4 Microarrays Unit, Josep Carreras Leukaemia Research Institute (IJC), 08916 Badalona, Spain

5 MDS Group, Josep Carreras Leukaemia Research Institute (IJC), 08916 Badalona, Spain

[†]These authors contributed equally to this paper as co-first authors

[#]Correspondence to: Marcus Buschbeck, email: mbuschbeck@carrerasresearch.org

Supplementary Figure S1

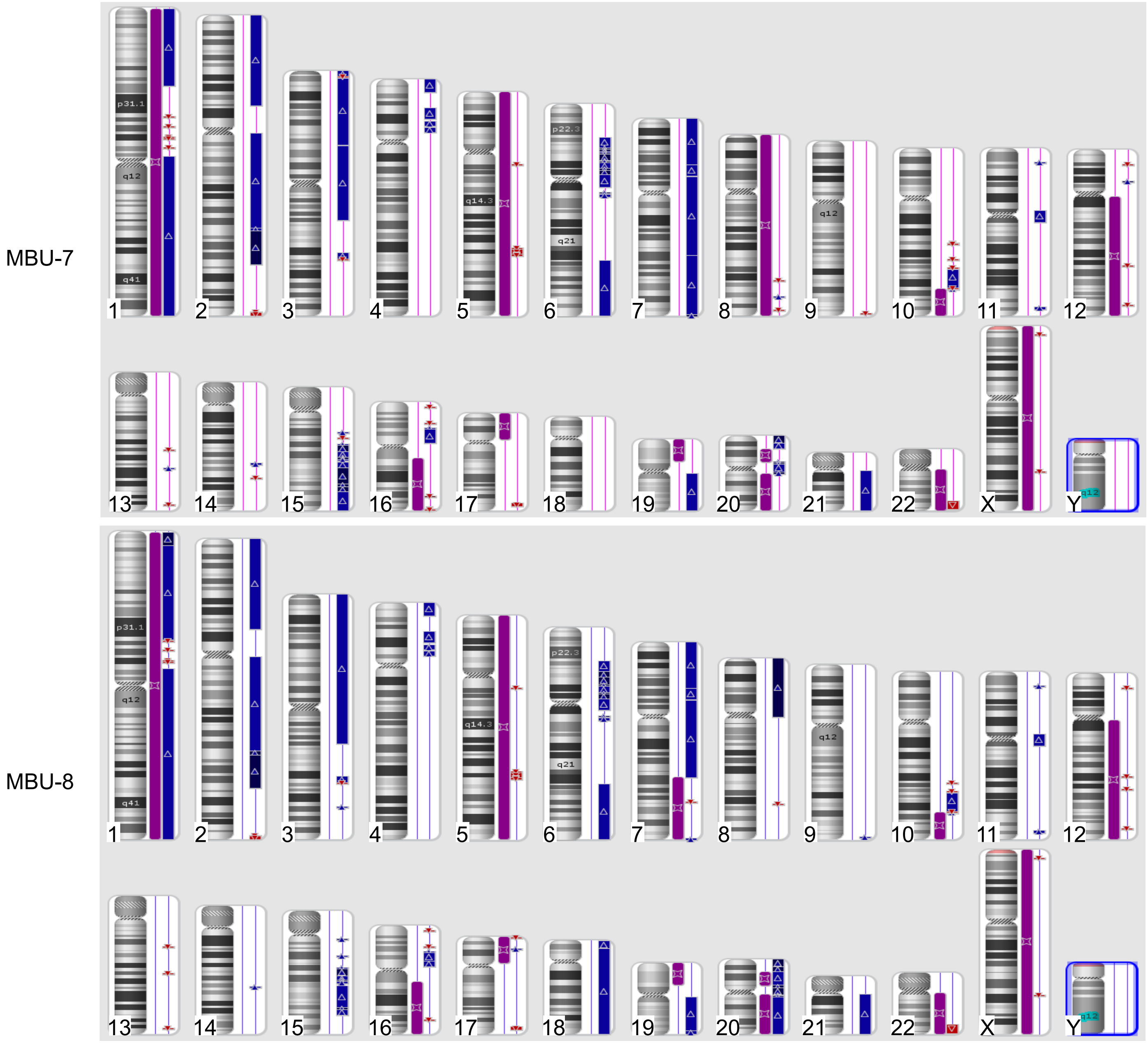

Figure S1. CytoScan 750k Array analysis of MBU-7 and MBU-8. Karyoview showing copy number variations (CNVs) (red bar, loss; blue bar, gain; purple, region of heterozygosity (ROH)) in MBU-7 and MBU-8. The size of each bar reflects the size of the CNV or ROH proportionally.

Supplementary Data S1: Karyotype formula of MBU-7 and MBU-8 as determined in the CytoScan array.

MBU-7

arr[GRCh37] 1p36.33q44(888659_249198164)x3
hmz,1p36.33p31.3(849467_63037530)x3,1p22.3p22.2(88226329_88863277)x1,1p21.3(95973102_96316702)x1,1p21.1(105822105_106075238)x1,1
p21.1(104532415_105039557)x1,1p13.2(113074253_113185886)x1,1p12q44(120523496_249224684)x3,2p25.3p13.2(12771_73262505)x3,2q11.1q
31.1(95327874_172555082)x3,2q31.1(172558454_174764944)x21,2q31.1q33.1(174768590_201529805)x4,2q37.3(240117667_240363438)x0,2q37.
3(240383204_242782258)x1,3p26.3p26.1(61892_4609648)x3,3p26.1(4614185_4713511)x1,3p26.1p14.2(4880547_60178909)x3,3p14.2q13.33(606
06230_120721775)x3,3q24q25.1(147270658_152023067)x3,3q25.1q25.2(152023446_152130477)x1,4p16.3p16.1(68346_10542148)x3,4p15.2p15.1
(23433730_32372969)x3,4p15.1p14(33908446_37519560)x3,4p14p13(39141343_43004684)x3,5p15.33q35.3(113577_180692321)x2
hmz,5q11.2q12.1(58663831_59012928)x1,5q23.2(125961992_126169850)x1,5q23.2q31.1(126988749_130990841)x1,5q31.1(130995734_13111497
9)x0,5q31.1(131140241_132533349)x1,6p22.1p21.31(27451555_35717146)x3,6p21.31p21.2(35720418_36656709)x4-
5,6p21.2(36661533_38805709)x4,6p21.2(38812390_39568268)x3,6p21.2p21.1(39575892_40911441)x4,6p21.1(40916956_45931289)x7,6p21.1p12.
3(45936019_46508141)x3,6p12.3(46515239_47408748)x4,6p12.3p12.2(47410560_52422170)x3,6p12.2p12.1(52430523_53903170)x5-
6,6p12.1p11.2(53907910_57117829)x4,6p11.2q12(57130227_66803535)x3, 6q13(71196270_71585762)x3-
4,6q13(73352418_75507700)x3,6q22.32q27(126324120_170914297)x3,7p22.3p14.2(43377_37167180)x3,7p14.1p12.3(37441667_46241652)x3,7p1
2.3q31.1(46912898_110399009)x3,7q31.1q36.3(110599806_157928710)x3,7q36.3(157945798_159119707)x4,8p23.3q24.3(168484_146292734)x2
hmz,8q24.11(117759023_117869800)x1,8q24.21(130444511_130677646)x3,8q24.3(141257264_141617376)x1,9q34.3(139286674_139543225)x1,1
0q22.2(76745048_76844206)x1,10q22.2(77171192_77409571)x1,10q23.31(89651856_89708904)x1,10q23.33(96495106_96739216)x1,10q23.33q2
5.2(96741497_112947554)x3,10q25.2(112954802_113425153)x1,10q25.2(113442694_114202495)x3,10q25.2q26.3(113564630_135426384)x2
hmz,11p15.3(11011041_11179251)x2-
3,11p11.12q12.1(50306193_59598423)x3,11q24.2q24.3(127665568_129446111)x3,12p13.2(11856946_12045096)x1,12p12.1(25320947_25481682
)x4,12q11q24.33(38190103_133777562)x2
hmz,12q22(93590431_93778522)x1,12q24.31(125083696_125231569)x1,13q21.31(64425283_65144143)x1,13q31.1(79361149_79795716)x3,13q3
3.3q34(110016342_110413026)x1,14q23.3q24.1(67684731_68894360)x3,14q31.1(79652578_79931998)x1,15q14(37304133_38306173)x3,15q15.1
(42028961_42129499)x1,
15q21.1(47335670_48293844)x3,15q21.1q21.3(48299651_53796191)x4,15q21.3(53797223_57996512)x3,15q21.3(58000388_58253062)x4,15q21.3
q22.2(58256571_59755507)x4,15q22.2(59886555_60074960)x3,15q22.2q22.31(62198797_66871214)x3,15q22.31q25.1(66878271_80728567)x4,15
q25.1q25.2(80734542_82574722)x5,15q25.2q25.3(82580674_86626153)x4,15q25.3q26.3(86637304_102399536)x3,16p13.3(3668923_3951551)x1,
16p12.3(17409998_17543397)x1,16p12.2(21405328_22306274)x3,16p12.2p11.2(22710614_34011784)x3,16q11.2q24.3(46504467_90146366)x2
hmz,16q23.1(78247738_78469453)x1,16q23.1(78474407_78560516)x0,16q24.3(88706233_88875928)x1,16q24.3(89455937_89481546)x1,17p13.3
p11.2(18901_22170994)x2 hmz,17q25.2q25.3(74903882_77352221)x1,19p13.3p13.11(260912_18809694)x2
hmz,19q11q13.43(28271120_58956816)x3,20p11.21q11.21(24207224_30596364)x3,20p12.3p12.2(6841717_11032787)x3,20p12.2p11.22(1106423
7_22198138)x2
hmz,20p11.22p11.21(21914364_22904906)x5,20p11.21(22910827_24196578)x4,20p11.21q11.21(24207224_30596364)x3,20p11.21q11.21(242072
24_30596364)x3,20q11.21q13.33(31859952_62912463)x2
hmz,21q11.2q22.3(15016487_48093361)x3,22q11.1q13.33(16888900_51157531)x2,hmz,22q13.2q13.33(43241043_50250253)x1,Xp22.33q28(1699
22_155233846)x2 hmz,Xp22.31(7065205_7162603)x1,Xq25(122575508_122634218)x1

MBU-8

arr[GRCh37] 1p36.33p36.22(849467_11993635)x4,1p36.33q44(888659_249198164)x3
hmz,1p36.22p22.3(12018792_88086538)x3,1p22.3p22.2(88279171_88863277)x1,1p21.3(95973102_96341350)x1,1p21.1(104532415_105039557)x
1,1p21.1(105822105_106075238)x1,1p13.3q44(111418802_249224684)x3,2q37.3(240343659_242782258)x1,2q37.3(240123898_240337744)x0,2q
31.1q33.1(174983004_201355668)x4,2q31.1(172014778_174981749)x22,2q11.1q31.1(95327874_172008974)x3,2p25.3p13.2(12771_73279605)x3,
3p26.3q13.33(61892_120763112)x3,3q24q25.1(147270658_151910401)x3,3q25.1q25.2(152032655_152130477)x1,3q26.31(171519730_171700771
)x3,3p16.3p16.1(68346_10551560)x3,5p15.33q35.3(113577_180692321)x2
hmz,4p15.2p15.1(23433730_32357667)x3,4p15.1p14(33907346_37519560)x3,4p14p13(39141343_43004684)x3,5q11.2(58663909_58878845)x1,5q
23.2(125957275_126169850)x1,5q23.2q31.1(126988749_130950492)x1,5q31.1(130967948_131076638)x0,5q31.1(131082267_132505660)x1,6p22.
1p21.31(27459319_35539822)x3,6p21.31p21.2(35544800_40440322)x4,6p21.2p12.3(40441630_46454002)x7,6p12.3p12.2(47439730_52636851)x3
,6p12.3(46460879_47430456)x4,6p12.2p12.1(52653439_54114446)x6,6p12.1p11.2(54118476_57713366)x4,6p11.2q12(57751039_66809901)x3,6q
13(71192813_71585762)x4,6q13(73352418_75507700)x3,6q22.32q27(126324120_170914297)x3,7p22.3p14.2(43377_37173048)x3,7p14.1p12.3(3
7441667_46241652)x3,7p12.3q31.1(46912898_108984780)x3,7q31.1q36.3(108918095_159118443)x2
hmz,7q32.1(128564357_128843282)x1,7q36.3(157945798_159119707)x3,8p23.3q11.1(158049_47644478)x4,8q24.11(117759023_117850398)x1,9
q34.3(139367119_139850030)x3,10q23.31(89651619_89708903)x1,10q23.33(96476363_96739216)x1,10q23.33q25.2(96741497_112947554)x3,10
q25.2(112954802_113425153)x1,10q25.2(113442694_114195645)x3,10q25.2q26.3(113585012_135426384)x2
hmz,11p11.12q12.1(50306193_59598423)x3,11p15.3(11019469_11179251)x3,11q24.2q24.3(127646077_129446111)x3,12p13.2(11856946_120337
70)x1,12q11q24.33(38190103_133777562)x2
hmz,12q21.31(83133524_83254062)x1,12q22(93590431_93784506)x1,12q24.31(125083696_125231569)x1,13q14.11(42184928_42787040)x1,13q
21.31(64425283_65144143)x1,13q33.3q34(110016342_110419404)x1,14q23.3q24.1(67728391_68894360)x3,15q11.2(22935761_23288350)x4,15q
14(37304578_38285599)x3,15q21.1(47338812_48362283)x3,15q21.1q21.3(48372378_53930583)x4,15q21.3(53934735_57867081)x3,15q21.3(578
74019_58536395)x4,15q21.3q22.2(58577163_60074960)x3,15q22.2q25.1(62198797_80728567)x3,15q25.1q25.2(80734542_82169097)x5,15q25.2q
25.3(82180114_86637304)x3,16p13.3(3668923_3927847)x1,16p12.3(17409998_17543397)x1,16p12.2(21379629_22312591)x3,16p12.2p11.2(2271
0614_29426399)x3,16p11.2(29769994_34011784)x3,16q11.2q24.3(46504467_90146366)x2
hmz,16q23.1(78247738_78551936)x1,17p13.3p11.2(18901_22170994)x2
hmz,17p13.3(284265_678478)x1,17p13.1(10014100_10402323)x3,17q25.2q25.3(74903882_77352221)x1,18p11.32p11.31(136228_6723883)x3,18p
11.31q23(6743814_78013728)x3,19p13.3p13.11(260912_18809694)x2
hmz,19q11q13.42(28273345_56251696)x3,19q13.42q13.43(56273044_58956816)x3,20p13p12.3(61662_6783148)x5,20p12.3p12.2(6787196_10674
963)x4,20p12.2p11.22(10678649_21796031)x3,20p12.2p11.22(10694848_22271603)x3
hmz,20p11.22p11.21(21798992_24357649)x6,20p11.21q11.21(24365385_29959695)x4,20q11.21q13.33(29510307_62912463)x3
hmz,20q11.21(29969184_30851155)x6,20q11.21q11.22(30856271_32137999)x5,20q11.22q13.33(32140327_62913645)x3,21q11.2q22.3(15016487
_48093361)x3,22q11.1q13.33(16888900_51157531)x2
hmz,22q13.2q13.33(43230266_50262454)x1,Xp22.31(7051930_7162603)x1,Xp22.33q28(169922_155233846)x2
hmz,Xq25(122575508_122634218)x1

Supplementary Table S2: Antibodies used for immunophenotypic analysis.

Surface Marker	Fluorochrome	Catalogue number Beckman Coulter
CD15	FITC	B36298
CD10	APC	B92400
CD117	PC7	B49221
CD11b	AA750	B36295
CD123	PE	B14808
CD13	PE	A07762
CD33	PC5.5	B36289
CD34	AA700	B92417
CD38	FITC	A07778
CD4	PE	A07751
CD44	AA750	B30637
CD45	KrO	B36294
CD56	PC5.5	B49189
CD64	ECD	A98434
CD99	ECD	B76291
HLA-DR	PB	B36291
